# Clinical presentation and angiographic findings of acute myocardial infarction in young adults in Jazan region

**DOI:** 10.1186/s12872-023-03335-3

**Published:** 2023-06-16

**Authors:** Kamel H. Haider, Sultan Abdulwadoud Alshoabi, Ibrahim A. Alharbi, Moawia Gameraddin, Osamah M. Abdulaal, Awadia Gareeballah, Walaa M. Alsharif, Fahad H. Alhazmi, Abdualziz A. Qurashi, Khalid M. Aloufi, Ahmed I. Sayed

**Affiliations:** 1 Cardiology Department, Cardiac Center, Prince Mohammed Bin Nasser Hospital, Jazan, Kingdom of Saudi Arabia; 2 Department of Diagnostic Radiology Technology, College of Applied Medical Sciences, Taibah University, Almadinah Almunawwarah, Kingdom of Saudi Arabia; 3 Internal Medicine Department, University of Jazan, Jazan, Kingdom of Saudi Arabia

**Keywords:** Smoking, Obesity, Sedentary lifestyle, Khat chewing, Left anterior descending artery (LAD), Right coronary artery (RCA), Left circumflex artery (LCX)

## Abstract

**Background:**

There is a paucity of information about the clinical features and angiographic findings in young patients with acute myocardial infarction (MI), especially in the Arab Peninsula countries.

**Objective:**

The aim of this study was to assess the proposed risk factors, clinical presentation, and angiographic findings of acute myocardial infarction in young adults.

**Methods:**

This prospective study included young (range, 18 to 45 years) patients who presented with acute MI based on clinical evaluation, laboratory investigation, and electrocardiogram, and they underwent a coronary angiography procedure.

**Key findings:**

Data of 109 patients with a diagnosis of acute MI were collected. Patients’ mean age was 39.98 ± 7.52 years (range, 31 to 45 years), and 92.7% (101) were male. Smoking was the highest risk factor in 67% of patients, obesity and overweight in 66%, sedentary lifestyle in 64%, dyslipidaemia in 33%, and hypertension in 28%. Smoking was the most common risk factor for acute MI in males (*p* = 0.009), whereas sedentary lifestyle was the most common risk factor in females (*p* = 0.028). Chest pain typical of acute MI was the most common presenting symptom in 96% of patients (*p* < 0.001). On admission, 96% of patients were conscious, and 95% were oriented. On angiography, the left anterior descending artery (LAD) was affected in 57%, the right coronary artery (RCA) was affected in 42%, and the left circumflex artery (LCX) was affected in 32% of patients. The LAD was severely affected in 44%, the RCA was severely affected in 25.7%, and the LCX was severely affected in 19.26% of patients (*p* < 0.001).

**Conclusion:**

Smoking, obesity, sedentary lifestyle, dyslipidaemia, and hypertension were the most common risk factors for acute MI. Smoking was the most common risk factor in males and sedentary lifestyle in females. The LAD was the most commonly affected coronary artery, followed by the RCA and LCX arteries, with the same order for severity of stenosis.

## Introduction

Myocardial infarction (MI) is defined as a myocardial injury that involves cell death due to prolonged ischemia as a result of an imbalance between the oxygen (O2) supply and demand of the myocardial wall. Acute MI occurs mostly due to insufficient blood flow to the myocardial tissue caused by atherothrombotic coronary artery disease (CAD), coronary artery embolism, and coronary spasm, as well as sustained tachyarrhythmias, severe blood loss, severe anaemia, or respiratory failure [1]. Acute MI is a common disease and remains a leading cause of morbidity and mortality worldwide. Although MI is a disease of old age, recent studies found that the incidence of MI is 12.9 and 38.2 per 1,000 in males and 2.2 and 5.2 per 1,000 in females in the age groups of 30–34 and 35–44 years, respectively [2]. Diabetes mellitus (DM), depression, hypertension, smoking, a family history of MI, low household income, hypercholesterolemia, obesity, sedentary life, previous MI, and previous percutaneous coronary intervention (PCI) are the risk factors of acute MI in young male and female patients [2, 3].

Diagnosis of acute MI can be made based on clinical presentation, cardiac biomarkers, and electrocardiogram (ECG). In young adults, a clinical presentation of acute MI is similar to that of older patients, and most patients present with chest pain. Due to a lower suspicion of acute MI and atypical presentation in some patients, there is more likely to be a delay in diagnosis of acute MI in young adults [4, 5]. Cardiac troponin I (cTnI) and cardiac troponin T (cTnT) are components of the contractile apparatus of myocardial cells, which are expressed almost exclusively in the heart. Elevated cTnI values occur exclusively in an injury to cardiac tissues. Both cTnI and cTnT are the preferred biomarkers for evaluation of myocardial injury and are recommended for routine use [6].

ECG is a pivotal tool in the diagnosis of acute MI, and it can be used to determine the presence, location, and extent of MI in acute coronary occlusion. ECG manifestations that suggest acute MI, in the absence of left ventricular hypertrophy or bundle branch block, are as follows: (1) ST elevation, which is the presence of new ST elevation at the J-point in two contiguous leads with the cut-point of 1 mm in all leads other than leads V2–V3, where the following cut-points apply: > 2 mm in males > 40 years, > 2.5 mm in males < 40 years, or > 1.5 mm in females regardless of age; (2) ST depression and T-wave changes: the presence of new horizontal or down-sloping ST depression > 0.5 mm in two contiguous leads and/or T inversion > 1 mm in two contiguous leads with prominent R wave or R/S ratio > 1 [6, 7].

Acute MI occurs after critical or total occlusion of the coronary arteries, mostly due to thrombosis or atherosclerotic plaque rupture or erosion, and manifests with a similar clinical presentation. However, determining acute MI type and understanding the similarities and differences between ST-elevation MI (STEMI) and non-ST-elevation MI (NSTEMI) is an essential step for proper management [8]. STEMI is defined as acute coronary thrombosis or persistent ST-segment elevation of ≥ 1 mm in ≥ 2 contiguous ECG leads. NSTEMI is defined as ischemic symptoms at rest, lasting ≥ 10 min, which arises from an acute coronary plaque rupture or erosion, occurring within 24 h before hospital admission and displaying either elevated creatinine kinase or cardiac troponin (cTn) cardiac biomarkers within 24 h of the initial clinical presentation [9].

Despite the relatively low prevalence of acute MI in young adults, the potential of death and long-term disability makes it a highly significant clinical problem. In the literature, there is a paucity of information about the clinical features and angiographic findings in young patients with acute MI, especially in the Arab Peninsula countries. The aim of this study was to assess the proposed risk factors, clinical presentation, and angiographic findings of acute MI in young adults, who were defined as patients under 45 years old. The significance of this study lies in highlighting the significant proportion of acute MI patients with modifiable risk factors who could benefit from more intensive prevention strategies.

## Patients and methods

### Study design

This observational prospective study was conducted from November 2021 to September 2022 at Prince Mohammed bin Nasser Hospital (PMBNH) Cardiac Center in Jazan Region, Southern Province, of Kingdom of Saudi Arabia (KSA). Patients presented or referred to the centre underwent clinical evaluation, laboratory investigation of cardiac biomarkers, ECG, echocardiogram (echo), and coronary angiography for diagnostic and therapeutic purposes. The data of these patients were collected for this study, and the inclusion criteria were the following: each young patient who presented to PMBNH, Jazan, with acute MI, either STEMI or NSTEMI, and for whom informed consent was obtained to enrol in this study.

Exclusion criteria were as follows: patients with acute MI older than 45 years old, patients with acute MI younger than 18 years old, and patients with other heart diseases. Patients’ information on clinical presentation, risk factors, ECG changes, echo, and angiographic findings were collected by using a questionnaire filled out for each patient.

### Diagnosis of MI

In this study, patients were diagnosed with MI based on clinical symptoms, signs, ECG findings, and cardiac biomarker values, and they underwent coronary angiography with possible PCI at the same centre.

### Statistical analysis

The collected data were analysed using the Statistical Package for Social Sciences (SPSS) (IBM Corp., Armonk, NY, USA). Categorical variables were reported as numbers and percentages, and continuous variables were reported as means ± standard deviation. A binomial test was used to present the variables. Binary logistic regression was performed to determine the correlation between acute MI and the proposed risk factors. The receiver operator characteristic (ROC) curve was applied to study the sensitivity and specificity of clinical presentation to diagnose acute MI.

## Results

For this study, the data of 109 young patients with a diagnosis of acute MI were collected. Patients’ mean age was 39.98 ± 7.52 years (range, 31 to 45 years); 92.7% (101) were male, and 7.3% (8) were female. The sociodemographic data of the enrolled patients in this study are provided in Table 1.

**Table 1:** Socio-demographic data of the participants

Variable	Measure/Level	No. (%)
Age (years)	Mean (SD)	39.98 ± 7.52
Gender	Male	101 (93%)
	Female	8 (7%)
Ethnicity	Arabian	86 (78.9%)
	South Asian	23 (21.1%)
Nationality	Saudi	75 (69%)
	Non-Saudi	34 (31%)
Body mass index (BMI)	Mean (SD)	26.12 ± 6.81

Smoking was a risk factor in 67% of patients, obesity and overweight in 66%, sedentary lifestyle in 64%, dyslipidaemia in 33%, hypertension in 28%, family history of CAD in 14%, previous history of MI in 6%, and history of previous PCI in 4% (Table 2).

**Table 2:** Myocardial infarction (MI) risk factors

Variable	Measure/Level	No. (%)	Statistical hypothesis testing	*P-value*
**Smoking**	Yes	73 (67%)	Binomial test	0.001
	No	36 (33%)		
**Obesity and overweight**	BMI ≥ 25 kg/m^2^	72 (66%)	Binomial test	0.001
	BMI < 25 kg/m^2^	37 (34%)		
**Sedentary life**	Yes	70 (64%)	Binomial test	0.004
	No	39 (36%)		
Hypertension	Yes	30 (28%)	Binomial test	< 0.001
	No	79 (72%)		
Diabetes mellitus (DM)	Yes	41 (38%)	Binomial test	0.012
	No	68 (62%)		
Dyslipidemia	Yes	36 (33%)	Binomial test	0.001
	No	73 (67%)		
Family history of CAD	Yes	15 (14%)	Binomial test	< 0.001
	No	94 (86%)		
History of MI	Yes	6 (6%)	Binomial test	< 0.001
	No	103 (94%)		
History of PCI	Yes	4 (4%)	Binomial test	< 0.001
	No	105 (96%)		

*BMI* Body mass index, *DM* Diabetes mellitus, *kg* Kilogram, *M* Meter, % Percentage, *CAD* Coronary artery disease, *MI* Myocardial infarction, *PCI* Percutaneous coronary intervention

Chest pain typical of MI was the presenting symptom in 96% of patients (OR = 8.634, 95% CI 1.047–71.218), followed by shortness of breath (45%), atypical pain (8%), cardiac arrest (4%), syncope (3%), and palpitation (2%) (Table 3).

**Table 3:** Presented symptoms of myocardial infarction (MI) patients

Variable	Measure/Level	No. (%)	Statistical hypothesis testing	*P-value*
**Typical chest pain**	Yes	105 (96%)	Binomial test	< 0.001
	No	4 (4%)		
Epigastric/Shoulder/Back/Neck pain	Yes	9 (8%)	Binomial test	< 0.001
	No	100 (92%)		
Shortness of breath (SOB)	Yes	49 (45%)	Binomial test	0.338
	No	60 (55%)		
Syncope	Yes	3 (3%)	Binomial test	< 0.001
	No	106 (97%)		
Palpitation	Yes	2 (2%)	Binomial test	< 0.001
	No	107 (98%)		
Cardiac arrest	Yes	4 (4%)	Binomial test	< 0.001
	No	105 (96%)		

**Fig. 1:**
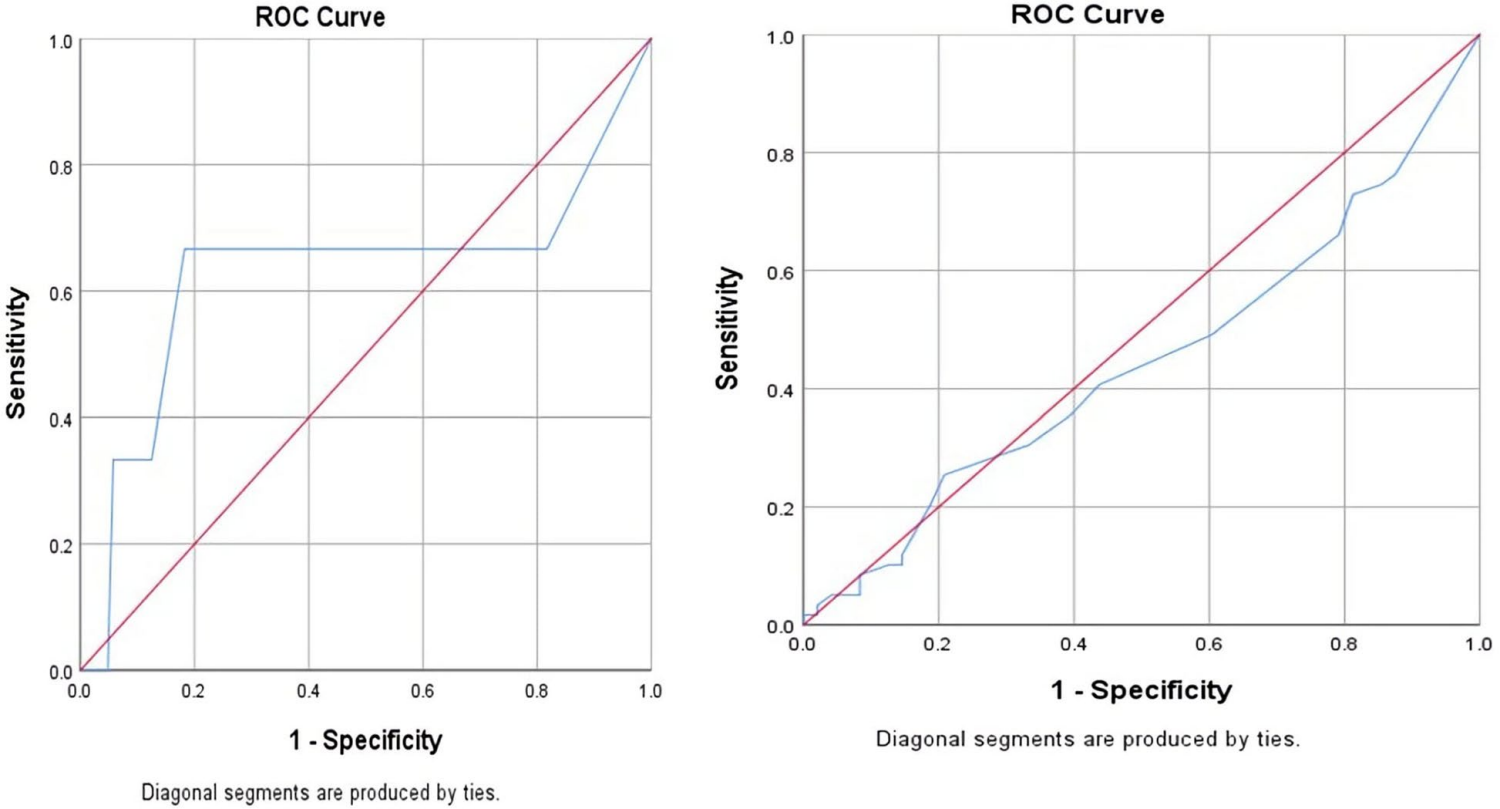
The receiver operator characteristic (ROC) curve shows significant sensitivity for typical chest pain as a presentation of acute myocardial infarction (MI) (area under the curve [AUC] = 0.681). However, it reveals low sensitivity for shortness of breath as a presentation of acute MI (AUC = 0.446)

The ROC curve shows significant sensitivity for typical chest pain as a presentation of acute MI (area under the curve [AUC] = 0.681). However, it reveals low sensitivity for shortness of breath as a presentation of acute MI (AUC < 0.5) (Fig. 1).

On admission, 96% of patients were conscious, 95% were oriented, and 74% of cases were diagnosed with STEMI. However, mechanical ventilation, focal neurological signs, congestive heart failure (CHF), rales and high jugular venous pressure, and cardiogenic shock were rarely present (Table 4).

**Table 4:** Patient status on admission

Variable	Measure/Level	No. (%)
Type of MI	**STEMI**	74 (68%)
	NSTEMI	35 (32%)
Consciousness	**Yes**	105 (96%)
Orientation	**Yes**	104 (95%)
Mechanical ventilation	No	105 (96%)
CHF	No	107 (98%)
Cardiogenic shock	No	104 (96%)

*MI* Myocardial infarction, *STEMI* ST-elevation myocardial infarction, *NSTEMI* Non-ST-elevation myocardial infarction, *CHF* Congestive heart failure

Our study showed that STEMI was the most common type of acute MI, and the most common type of STEMI was anterior (34.86%), followed by inferior (25.7%) (Table 5).

**Table 5:** Types of acute MI in this study

Variable	Categories	Subtypes	No. (%)
Types of acute MI	STEMI	Anterior	38 (34.86%)
		Inferior	28 (25.7%)
		Lateral	4 (3.7%)
	NSTEMI		39 (35.8%)
Total			109 (100%)

*STEMI* ST-elevation myocardial infarction, *NSTEMI* Non-ST-elevation myocardial infarction

**Fig. 2:**
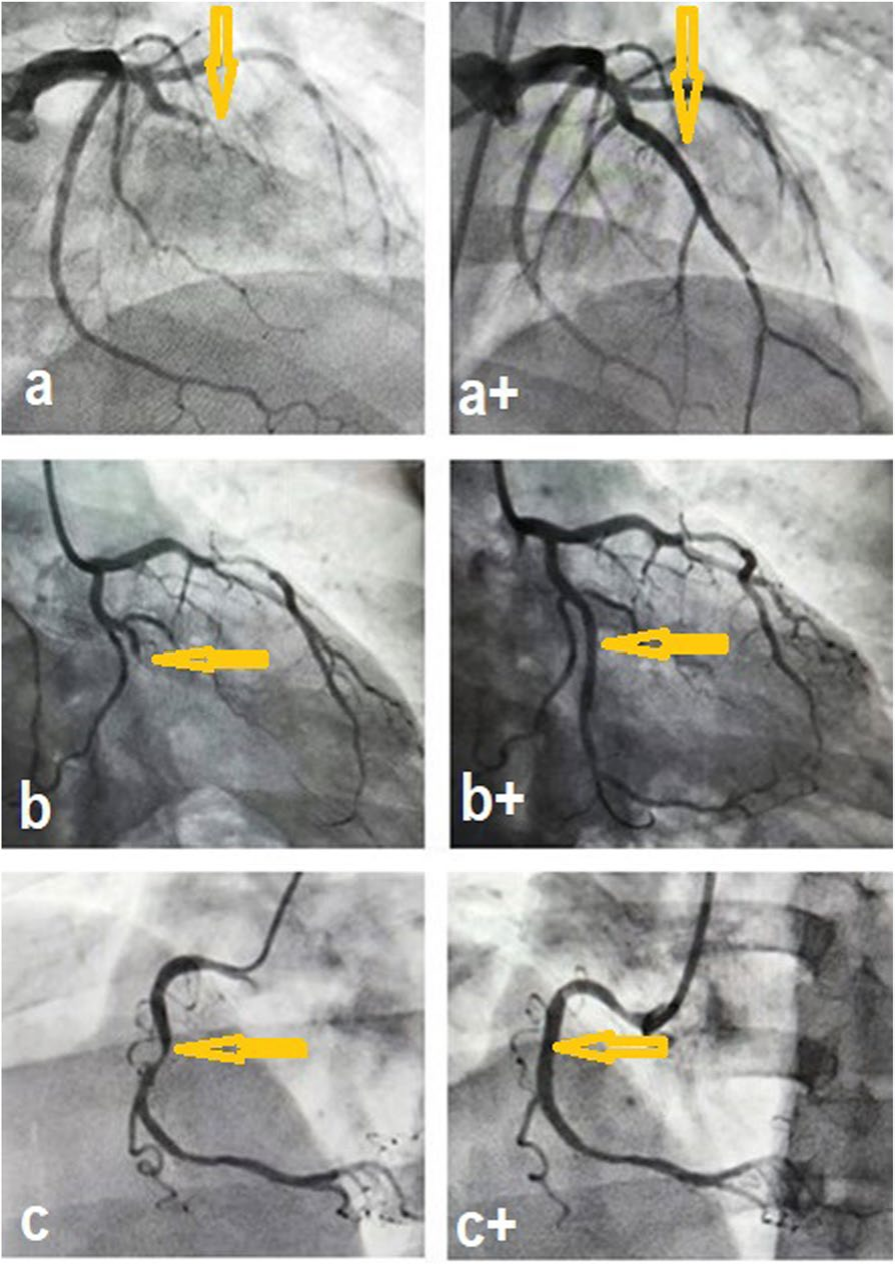
Angiographic images of **a**) a 41-year-old male patient presenting with acute anterior myocardial infarction (MI), with the images showing total occlusion of the proximal left anterior descending artery (LAD) (arrow) and, a +) after percutaneous coronary intervention (PCI), large LAD (arrow) that was occluded; **b**) a 42-year-old man presenting with posterior MI, with the coronary angiogram showing total occlusion of the left circumflex artery (LCX) (arrow) and b +) the left coronary angiogram after PCI showing patent LCX (arrow); **c**) a 38-year-old man presenting with non-ST elevation MI (NSTEMI), with the right coronary angiogram showing 80% stenosis in the mid-right coronary artery (RCA) (arrow) and c +) the right coronary angiogram after PCI showing complete resolution of the mid RCA stenosis (arrow)

On the angiography, the LAD was affected in 57% of patients, the RCA was affected in 42% of patients, the LCX was affected in 32% of patients, and other arteries were less likely to be affected (*p* < 0.001) (Table 6, Fig. 2).

**Table 6:** Angiographic findings of the patients

Variable	Measure/Level	No. (%)	Statistical hypothesis testing
Access	Radial	94 (86%)	Binomial test
	Femoral	15 (14%)	
Left main (LM) artery	Affected	4 (3.7%)	Binomial test
**Left anterior descending (LAD)**	Affected	62 (57%)	Binomial test
Diagonal artery	Affected	5 (4.6%)	Binomial test
Ramus artery	Affected	7 (6.4%)	Binomial test
**Left circumflex (LCX) artery**	Affected	35 (32%)	Binomial test
Obtuse marginal-1 (OM1)	Affected	14 (12.85%)	Binomial test
Obtuse marginal-2 (OM2)	Affected	1 (0.9%)	Binomial test
**Right coronary artery (RCA)**	Affected	46 (42.2%)	Binomial test
Posterior descending artery (PDA)	Affected	5 (4.6%)	Binomial test
Posterolateral branch (PLB)	Affected	3 (2.8%)	Binomial test
Number of the affected vessels	None	11 (10.4%)	Chi-square
	Single	68 (62.38%)	
	Two vessels	16 (14.67%)	
	Multiple vessels	14 (12.84%)

On the angiography, the LAD was severely affected in 44% of patients, the RCA was severely affected in 25.7% of patients, and the LCX was severely affected in 19.26% of patients (*p* < 0.001) (Table 7).

**Table 7:** The affected coronary arteries and degree of affection

Variable	Mild stenosis	Moderate stenosis	Severe stenosis or total occlusion	Total Number
LM artery	2 (1.8%)	1 (0.9%)	1 (0.9%)	4 (3.7%)
**LAD artery**	6 (5.5%)	8 (6.3%)	48 (44%)	62 (57%)
Diagonal artery	1 (0.9%)	2 (1.8%)	2 (1.8%)	5 (4.6%)
Ramus artery	0 (0.0%)	2 (1.8%)	5 (4.6%)	7 (6.4%)
**LCX artery**	8 (7.3%)	6 (5.5%)	21 (19.26%)	35 (32%)
OM1 artery	2 (1.8%)	3 (2.8%)	11 (10%)	14 (12.85%)
OM2 artery	0 (0.0%)	0 (0.0%)	1 (0.9%)	1 (0.9%)
**RCA**	5 (4.6%)	13 (11.9%)	28 (25.7%)	46 (42.2%)
PDA	0 (0.0%)	2 (1.8%)	3 (2.8%)	5 (4.6%)
PLB	0 (0.0%)	1 (0.9%)	2 (1.8%)	3 (2.8%)

*LM* Left main, *LAD* Left anterior descending artery, *LCX* Left circumflex artery, *OM1* Occipitomarginal-1, *OM2* Occipitomarginal-2, *RCA* Right coronary artery, *PDA* Posterior descending artery, *PLB* Posterolateral branch

Table 8 shows that 33.9% of PCIs were routine, 22.9% were primary, and 19.3% were rescue (*p* = 0.044).

**Table 8:** Types of percutaneous coronary intervention (PCI)

Variable	Categories	No. (%)	Statistical hypothesis testing	*P-value*
Type of PCI	No PCI	26 (23.85%)	Chi-square	0.044
	Primary	25 (22.9%)		
	Rescue	21 (19.3%)		
	Routine	37 (33.9%)		

## Discussion

Acute MI occurs primarily in older patients. However, young adults can be affected, which constitutes an important diagnostic problem for physicians. Many previous studies have used 40 to 45 years as an age cut-off to define young patients with acute MI [4]. In this study, we used 45 years old as a cut-off to define young patients, and we collected the data of 109 patients diagnosed with acute MI who underwent diagnostic coronary angiography and possible PCI. We found that 93% of the affected patients were male. This result is in line with Bhardwaj et al., who reported that young adult acute MI occurs almost exclusively in males [10]. The low incidence of acute MI in young premenopausal females is related to the positive effect of female hormones on the patient’s lipid profile, vascular activity, and vascular endothelial function [11]. Another previous study reported that there are sex differences in risk factors for MI, and females who have MI are often postmenopausal [12]. In their previous cohort study on thousands of participants, Millet et al. reported that sex-specific associations between MI and risk factors declines with age, and the incidence of MI in females tends to be similar to that in males [13].

Regarding risk factors, the American Heart Association (AHA), in conjunction with the National Institute of Health (NIH) statistics related to heart disease, stroke, and cardiovascular disease (CVD) risk factors, reported a sharp increase in electronic cigarette use among adolescents between 2011 and 2020. Persons who are physically active are at a 25–40% lower risk of CVD mortality. Daily intake of 5 (versus 2) servings of fruit and vegetables is associated with 13% lower total mortality and 12% lower total CVD mortality. Higher intake of total fat and unsaturated fatty acids is associated with lower total mortality. Risk of ischemic stroke increases consistently with metabolic syndrome severity. The death rate attributable to high blood pressure increased 34.2% from 2009 to 2019 [14]. Our results indicated that smoking, obesity and overweight, and a sedentary lifestyle are highly significant risk factors for acute MI. This result is in line with Rathore et al., who reported that smoking is a strong risk factor for acute MI, increased body mass index is directly related to increased incidence of acute MI, and physical activity may contribute to reducing risk of CAD by 20–30% [15].

Our study indicated a significant association between MI and male smoker patients and a significant association between MI and female patients with a sedentary lifestyle. Similar to our study, Azab et al. reported that smoking is the second most significant risk factor in male patients, and hypertriglyceridemia is the second most significant risk factor in females [16]. Nyström et al. reported that hyperlipidaemia was the only traditional risk factor more frequent among females than males [17]. In contrast to our results, Ilic et al. reported that risk of acute MI increases with obesity and stressful life in male patients and with DM and menopause in female patients [12]. We explain that the discrepancy in risk factors associated with gender is attributed to the difference in habits in different societies and areas around the world. In their previous study, Nyström et al. reported that social factors, such as serious life events, strained economy, stress, depression, and sleep deprivation, were stronger potential risk factors for acute MI among females than among males [17]. In their previous case control study, Lu et al. reported that potential modifiable risk factors including DM, depression, hypertension, current smoking, low household income, and hypercholesterolemia accounted for 85% of the risk of acute MI in young adult male and female patients [18]. These risk factors can be controlled to decrease the incidence of acute MI in young adults.

The current study shows that typical chest pain is the predominant presenting symptom of acute MI. The ROC curve revealed that chest pain is the only reliable presenting symptom of acute MI. This result is consistent with several previous studies, including Nyström et al., who reported that the dominant prodromal symptom of acute MI is chest pain in both males and females [17]. Ferry et al. also reported that chest pain was the most common presenting symptom in both females and males (approximately 93%) diagnosed with type-1 MI [19]. Malik et al. reported that chest pain is the most common presenting complaint of acute MI, and it is classically described as a heavy chest pressure or squeezing, a burning feeling, or difficulty breathing. The pain often radiates to the left shoulder, neck, or arm. However, acute MI pain may be atypical and cause an overlap in cardiac as well as non-cardiac pain, which requires a vigilant history evaluation of chest pain parameters to overcome this dilemma [20]. In the current study, typical chest pain, shortness of breath (SOB), and epigastric/shoulder/back/neck pain were the three most common symptoms of acute MI. These symptoms are consistent to some extent, especially the first symptom, with those reported in a previous study conducted by Mahajan et al., who reported that chest pain or discomfort, SOB, and pain or discomfort in arms or shoulders were the most common presenting symptoms of acute MI, with 91.8%, 87%, and 85.7%, respectively [21].

In our study, all enrolled patients were primarily diagnosed with acute MI using clinical features, ECG, and cTn, especially troponin I. In addition to presenting clinical features, ECG is an important non-invasive diagnostic tool for patients with acute MI [22], with 95–97% specificity for diagnosis of MI [23]. Current guidelines recommend serial ECGs for persisting symptoms [22], which can increase ECG sensitivity [23]. Due to the fact that cardiac troponin is the laboratory biomarker of choice for detection of cardiac injury [24], troponin I, which is highly sensitive to exclude acute MI [25], was used to confirm the diagnosis of acute MI in all patients of the current study. Diagnoses of acute MI were made based on the facts reported in previous studies that strategies using a single troponin I measured with a high-sensitivity assay on admission, combined with a normal or low-risk ECG, can safely rule out acute MI [26, 27]. Furthermore, echo is a highly valuable tool in establishing the diagnosis of acute MI, and it gives substantial information regarding risk stratification, detection, and follow-up mechanical complications of acute MI [28]. Echo can generate images of the inside and outside of the heart and is used to assess cardiac wall motion, valve abnormalities, ischemic mitral regurgitation, and cardiac tamponade [23]. In the current study, echo provides good information about wall movements and ejection fraction in most patients of the study.

Ultimately, definitive acute MI requires coronary catheterization, which monitors both blockage and flow of blood through the coronary arteries and serves both diagnostic and therapeutic purposes. All of the patients in our study were undergoing coronary catheterization. The LAD, which is the largest branch of the left main coronary artery and carries oxygenated blood to the left side of the heart, was the most commonly affected, followed by the RCA then LCX. This result is consistent with a previous study conducted by Ahmed et al., who reported that the LAD is the most commonly affected in both STEMI and NSTEMI [29]. The significance of the LAD, RCA, and LCX lie in the fact that they are the three largest coronary arteries [30]. Occlusion of the LAD may cause ischemia in the anterior wall of the left ventricle (LV) and manifests as precordial ST-segment elevation on ECG [31]. Occlusion of the RCA may cause ischemia of the inferior wall of the LV with or without the right ventricle and manifests as ST-segment elevation in inferior wall leads (II, III, and aVF) [32, 33]; however, it is exceptional to show ST-segment elevation in V1 to V3 [33]. Occlusion of the LCX or one of its branches may cause lateral wall infarction [34], which may manifest as ST-segment elevation in I and aVL [35]. ECG can determine the culprit occluded artery with high sensitivity and acceptable specificity [36]. However, diagnostic angiography is still the cornerstone in determining the affected arteries and the extent and percentage of occlusion, as well as providing a chart for interventional therapy.

### Limitations

This study is limited in that it included a small sample size. The prevalence of other significant suspected risk factors, such as serious life events, the strained economy, stress, depression, and sleep deprivation, were not involved in this study because we could not get such information from the patients, and no accurate medical records about them were available in our region.

## Conclusion

In this study, smoking, obesity, sedentary life, dyslipidaemia, and hypertension were the most common risk factors of acute MI, with a significant association of acute MI with male gender and khat chewing. Smoking was the most common risk factor in males and sedentary lifestyle in females. Acute MI patients were almost always conscious and oriented on presentation. LAD, followed by RCA and LCX, was the most commonly affected coronary artery, with the same order in severity of stenosis.

## Data Availability

The datasets used/or generated during the current study are available from the corresponding author on reasonable request.
